# Supplementary material for: Structure of the Secretory Compartments in Goblet Cells in the Colon and Small Intestine
Source: Cells. 2025 Jul 31;14(15):1185. doi: 10.3390/cells14151185 (PMC12346421; doi:10.3390/cells14151185)
Supplement: Supplementary file 1 [file cells-14-01185-s001.zip › cells-3536479-supplementary.pdf]

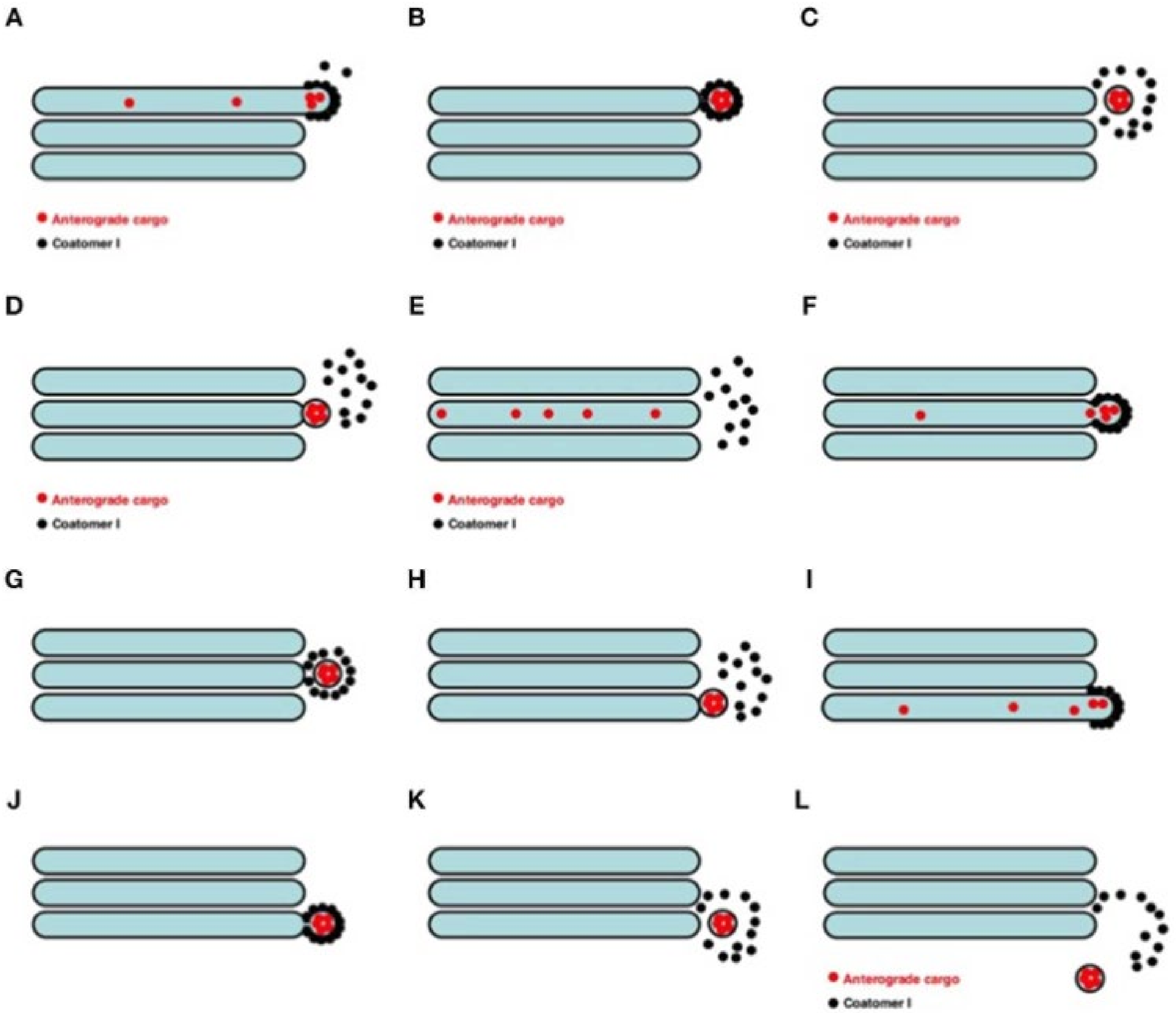

Figure S1. Scheme of the vesicular model of intra-Golgi transport. (A) COPI (black dots) forms a coat on a membrane bud. A cargo (red dots) is concentrated inside the COPI-coated bud. (B, C) This bud undergoes detachment (B) and uncoating (C). (D) The vesicle fuses with the distal Golgi compartment. (E) Distribution of the cargo within the next Golgi cisterna. (F–H and I–L) Repetition of the first stage. Finally, the vesicle can move out the Golgi (L). This image is taken from the Figure 5 presented paper by Mironov and Beznoussenko (2019[30]) in agreement with the Creative Commons License (reprinted courtesy Attribution–Non-commercial–Share Alike 4.0 Unported license).

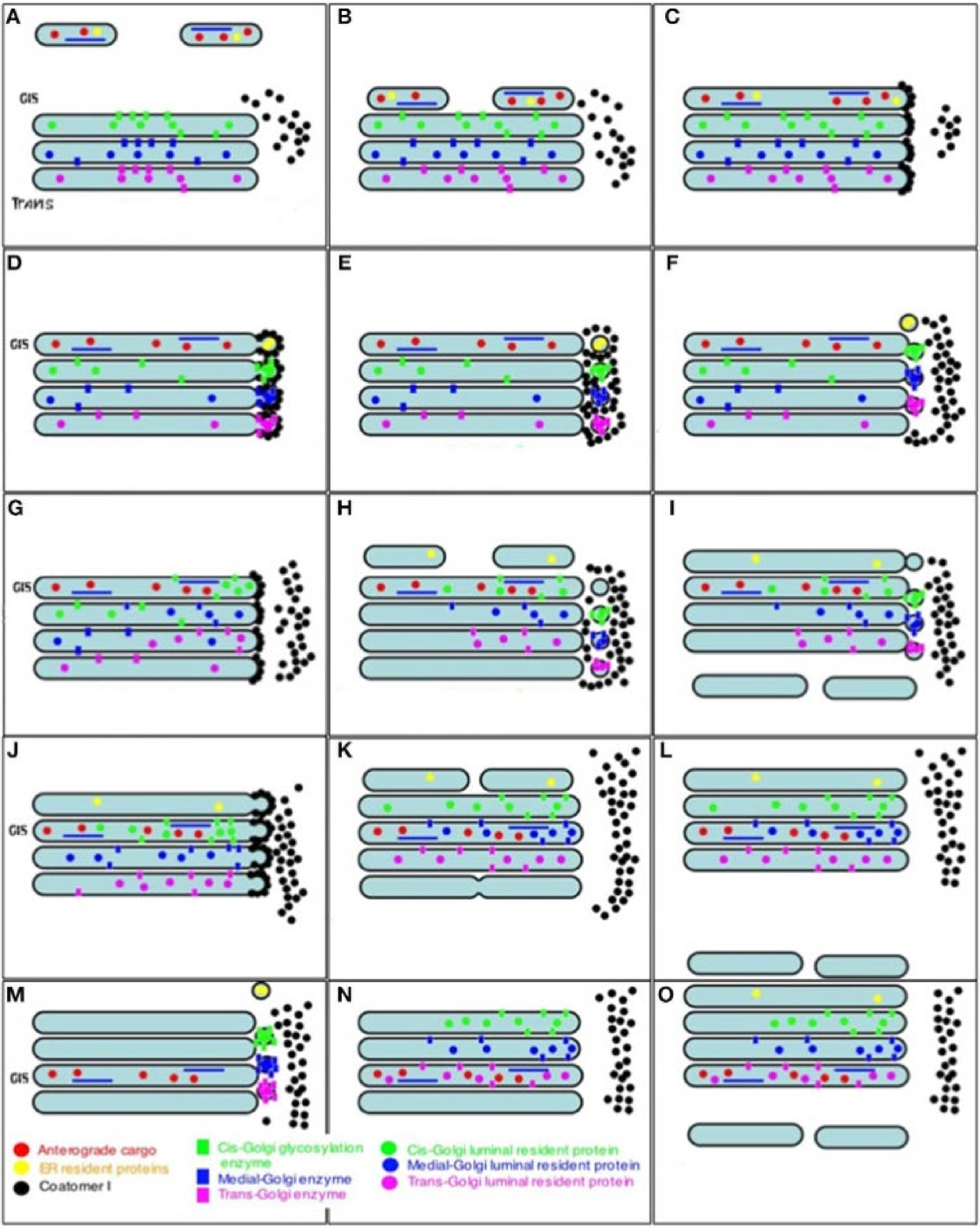

Figure S2. Scheme of intra-Golgi transport according to the CMPM. The main postulate of this model is that during intra-Golgi transport, the amount of cargo inside the cisterna during its progression is not changed, and that COPI vesicles (COPI, black dots; Golgi-resident proteins, coloured dots) should be concentrated in COPI vesicles. (A) Formation of ER-to-Golgi carriers (top). (B) Delivery of ER-to-Golgi carrier to the Golgi complex. (C) Fusion of ER-to-Golgi carriers and formation of the new *cis*-Golgi cisterna. (D) Formation of COPI-(black dots) coated buds on the Golgi

cisternae. (E) Detachment of buds and their uncoating. (F) COPI-dependent vesicles fuse with the proximal Golgi cisternae. (G–I) A new round of *cis*-cisterna formation, COPI-dependent budding, formation of vesicles, and their uncoating and fusion. (I) Departure of the most-trans cisterna in the form of post-Golgi carriers. (J–N) Additional rounds of similar events. (O) After step-wise departure of the post-Golgi carriers, the *cis*-Golgi cisterna formed after re-initiation of IGT becomes the *trans*-cisterna. This image is taken from the Figure 7 presented paper by Mironov and Beznoussenko (2019[30]) in agreement with the Creative Commons License (reprinted courtesy Attribution–Non-commercial–Share Alike 4.0 Unported license).

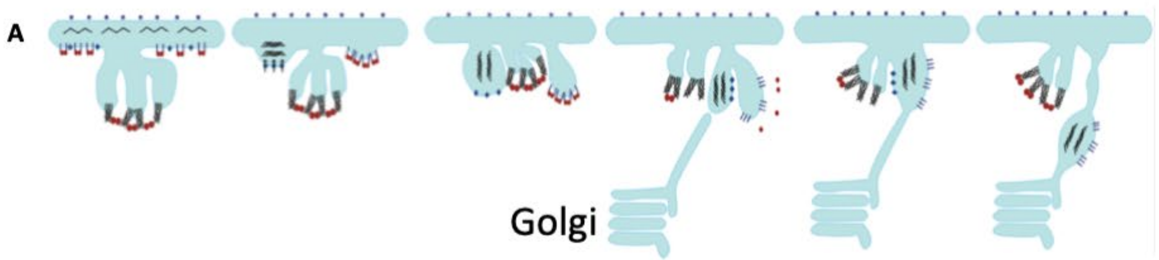
A
Golgi

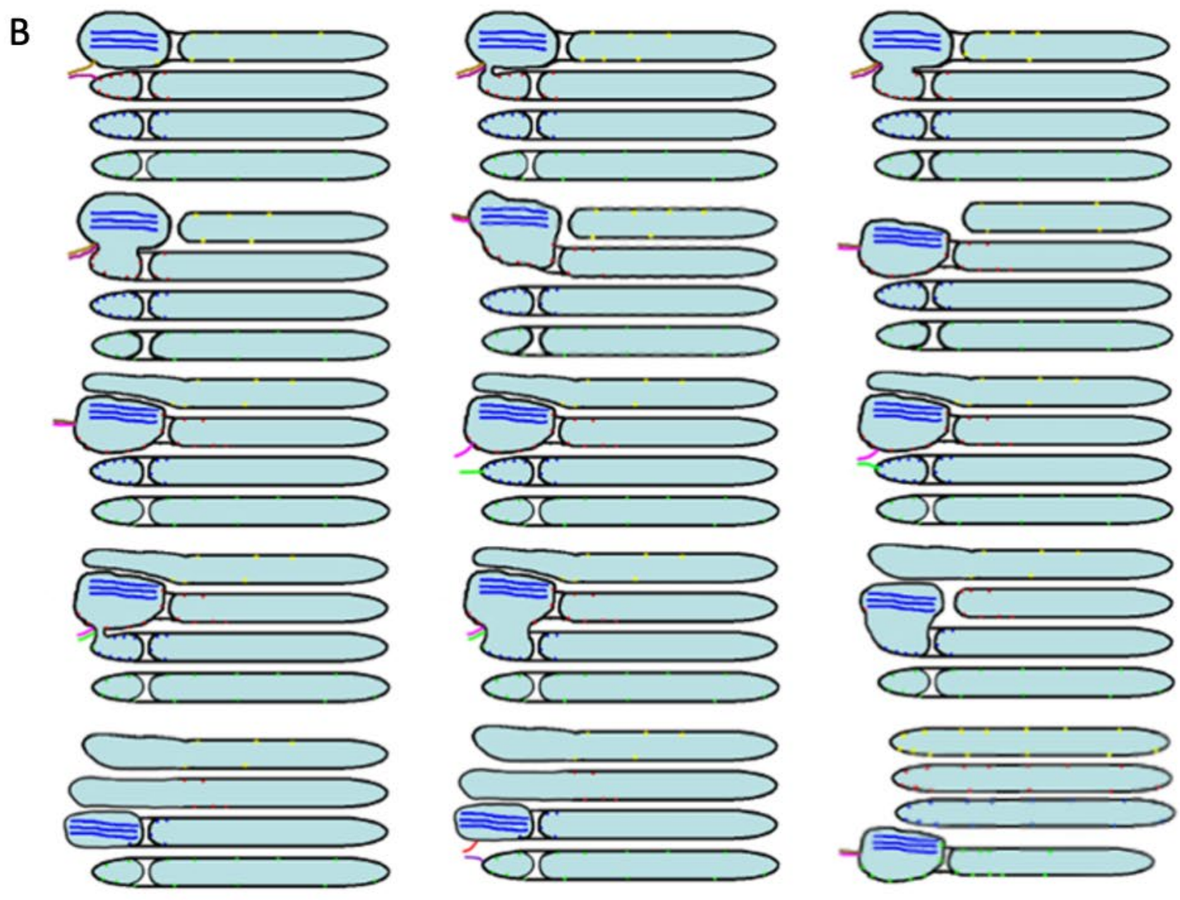
B

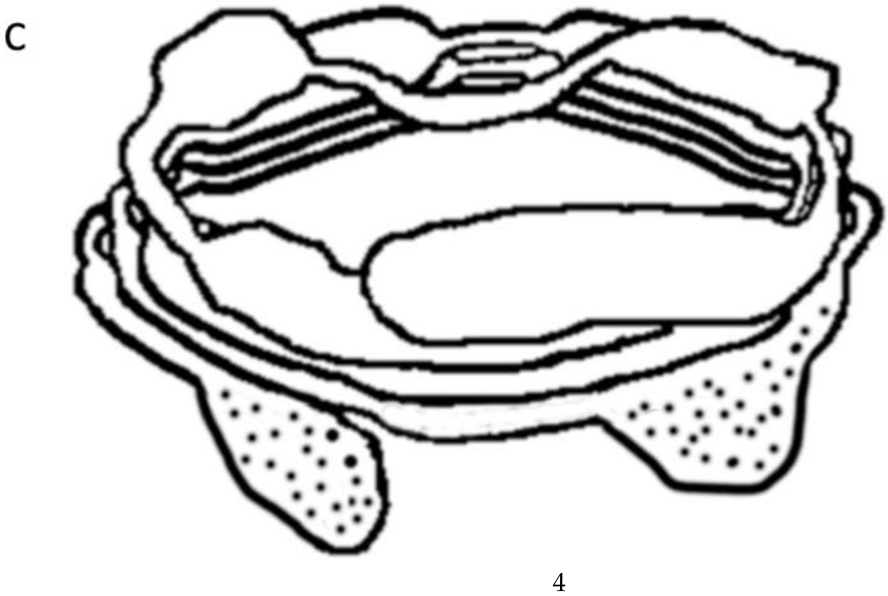
C

Figure S3. Scheme of the KARM at the level of ER-Golgi and intra-Golgi transport. (A) Initially the protrusion filled with mega-cargo is formed near the ERES coated with COPII. Then tubule from the Golgi arrives, fuses with the protrusion and next the distension is travelled as a bolus-like structure. (B) Distension of the cisterna fille with mega-cargo (blue lines) is moved from the cis-cisterna to the trans one according to initial fusion based on the use of SNAREs (Green and red lines) and subsequent fission. (C) Scheme of the spiral like Golgi able to realize the diffusion model. These images is taken from the paper by Mironov and Beznoussenko (2019[30]) in agreement with the Creative Commons License (reprinted courtesy Attribution–Non-commercial–Share Alike 4.0 Unported license).

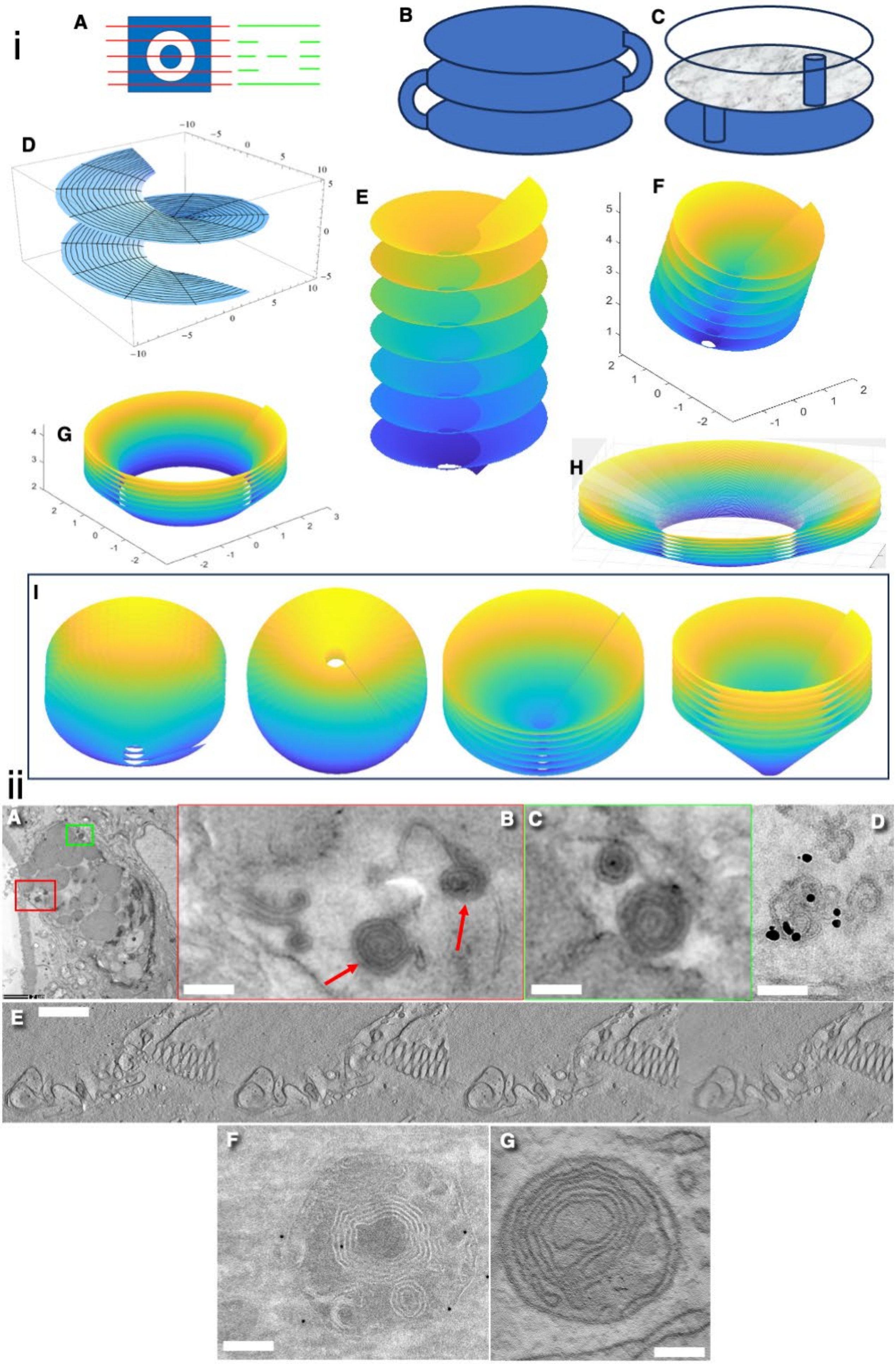
i
A
B
C
D
E
F
G
H
I
ii
A
B
C
D
E
F
G

Figure S4. i. Models of the Golgi organization. (A) Scheme explaining our strategy directed to find isolated Golgi stack. Blue colour shows the Golgi ribbon visible from the outside. Red line indicate sections. Green lines show the appearance of stack during sectioning. (B) The Golgi stack with tangential connections between rims. (C) Golgi stack with vertical connections between rims. (D-I) Different shape of the Golgi spiral based on the mathematical model explaining the formation of the spiral generated by a helicoid surface. (D) The helicoidal spiral without attachment of surfaces to each other (E) The spiral where layers of this surface are close to each other the hole in the center is small. (F, G) Spirals with large hole and different angle of the cone. (H) The cone with larger opening in the center and low sharpness. (I) Views of the spiral cone formed by helicoid surface with small hole in the center from different sides.

ii. Additional illustration of MLOs. (A) Goblet cells with MLOs in subapical zones. (B) Enlarged area inside the red box in (A). (C) Enlarged area inside the green box in (A). (D) Nanogold labelling of MLO for LC3. (E) Serial tomo-images (number 27, 39, 45, and 60 from the tomo-box) of the apical part of the goblet cell. (F) Immune EM labelling for LC3; cryosection. (G) Tomo-image. High magnification of MLO. Scale bars (nm): 220 (iA, iB); 500 (below C); 200 (iD); 2000 (E); 200 (F); 150 (G)
